# Intestinal Permeability Measured by Urinary Sucrose Excretion Correlates with Serum Zonulin and Faecal Calprotectin Concentrations in UC Patients in Remission

**DOI:** 10.1155/2019/2472754

**Published:** 2019-04-01

**Authors:** C. A. M. Wegh, N. M. de Roos, R. Hovenier, J. Meijerink, I. Besseling-van der Vaart, S. van Hemert, B. J. M. Witteman

**Affiliations:** ^1^Division of Human Nutrition, Wageningen University, Wageningen, Netherlands; ^2^Winclove Probiotics B.V., Amsterdam, Netherlands; ^3^Gastroenterology and Hepathology, Gelderse Vallei Hospital, Ede, Netherlands

## Abstract

**Background and Aims:**

Ulcerative colitis (UC) is associated with an increased intestinal permeability, possibly through a dysbiosis of intestinal bacteria. We investigated which markers are most relevant to assess intestinal permeability in UC patients and whether probiotics had an effect on these markers.

**Methods:**

In this twelve-week placebo-controlled randomized double-blind study, twenty-five subjects with UC in remission received either placebo or a multispecies probiotics. Samples of blood, urine, and faeces were taken at baseline, week 6, and week 12 to assess intestinal permeability and inflammation. Diaries and Bristol stool scale were kept to record stool frequency and consistency. Quality of life was scored from 32–224 with the inflammatory bowel disease questionnaire (IBD-Q).

**Results:**

This group of UC patients, in clinical remission, did not show increased intestinal permeability at baseline of this study. During the study, no significant group or time effects were found for intestinal permeability measured by the 5-sugar absorption test, serum zonulin, and faecal zonulin. Likewise, the inflammatory markers C-reactive protein (CRP), calprotectin, and the cytokines IFN*γ*, TNF*α*, IL-6, and IL-10 were not significantly affected. Stool frequency and consistency were not significantly affected either. The IBD-Q score, 194 for the probiotics group and 195 for the placebo group, remained unaffected. Correlations were tested between all outcomes; urinary sucrose excretion was significantly correlated with serum zonulin (*r* = 0.62) and faecal calprotectin (*r* = 0.55). Faecal zonulin was not significantly correlated with any of the other markers.

**Conclusion:**

Serum zonulin may be a more relevant biomarker of intestinal permeability than faecal zonulin, due to its correlation with other biomarkers of intestinal permeability. UC patients in remission did not show an effect of the probiotic treatment or a change in gut permeability. This should not discourage further studies because effects might be present during active disease or shortly after a flare up.

## 1. Introduction

The single-cell epithelial layer of the intestines has a dual function in digesting and absorbing nutrients and defending the body against the microorganisms and the pathogenic compounds in the gut [1, 2]. Different mechanisms protect this epithelial barrier [3]. Firstly, the epithelial cells are covered by a mucus layer, to protect the mucosal surface from harmful molecules and bacteria. Secondly, permeability of the intestinal epithelial cells is highly regulated by tight junctions and adherens junctions, which link the epithelial cells to each other. Also, the intestinal epithelial cells and immune cells in the intestine can recognize pathogens, release antimicrobial molecules, and secrete cytokines.

Improper functioning of the intestinal barrier function plays a central role in the pathogenesis of chronic intestinal inflammation, including ulcerative colitis (UC) [3]. UC is a chronic disease characterized by inflammation of the mucosal layer of the colon. Patients experience abdominal pain and bloody diarrhoea and have various gastrointestinal complaints even when in remission [4]. In the active disease state, mucosal inflammation is accompanied by impaired barrier function. Tight junctions are altered, and there is an increased incidence of apoptotic events. These barrier defects are attributed to enhanced activity of proinflammatory cytokines, which are highly expressed in the chronically inflamed intestine [5]. Whether changes in epithelial permeability in inflammatory bowel disease (IBD) patients play a primary role in disease pathogenesis or there is a secondary effect in response to inflammation is still under debate.

A possible intervention focussing on intestinal permeability in UC patients is the use of probiotics. Probiotics have been shown to be able to influence intestinal permeability both *in vitro* and *in vivo* [6–8]. Different probiotics, for instance, the mixture VSL#3, *Bifidobacterium longum* ssp. *longum* CCM 7952 and a lysate of the probiotic bacterium *Lactobacillus casei* DN-114001, prevented redistribution of tight junction proteins in a mouse model of dextran sulphate sodium-induced colitis [9–11].

In UC patients, different intervention studies with probiotics have been performed, to induce remission in active disease and preventing relapses in inactive disease [12, 13]. These studies have not investigated possible effects on epithelial barrier function. Overall, some of the intervention studies with probiotics have shown positive effects, but the total evidence is still limited, and further studies are necessary to get further insights into the aetiology of IBD and the working mechanisms of probiotics.

Increased intestinal permeability is usually assessed by sugar absorption tests such as the assessment of the lactulose/mannitol ratio in urine after ingestion of these sugars [14]. Recently, also other methods have been proposed [15]. One of these is the 5-sugar test, a relatively new absorption test that gives more information on site-specific changes in intestinal permeability than the formerly used dual sugar lactulose mannitol test [16]. The intestinal permeability of the gastroduodenal unit (sucrose/rhamnose ratio), small intestine (lactulose/rhamnose ratio), and the colon (sucralose/erythritol ratio) can be determined at the same time [14]. Another recent marker for gut permeability is the protein zonulin. Zonulin plays an important role in the disassembly of the tight junctions [2]. Increased zonulin concentrations are associated with an increase in intestinal permeability [17]. Zonulin can be measured in faeces and in serum, and although both are regarded as markers for intestinal permeability, results have not been consistent [18, 19].

As epithelial barrier defects in UC patients seem to be linked to inflammation, markers of inflammation might also be relevant to investigate these processes. C-reactive protein in blood serum is a valuable marker of disease activity in UC [20]. It is a general measure of inflammation, but not specific for inflammation in the intestinal tract. A sensitive marker for mucosal inflammation of the intestine is calprotectin, a leukocyte protein that is excreted in faeces [21]. Higher concentrations, above 100 *μ*g/g, are associated with a higher disease activity and are predictive for relapse of UC [22]. Lastly, cytokines are of interest due to their association with disease activity [23].

Currently, there is no consensus on how these different tests compare to one another and which ones are best as outcome parameters in intervention studies. Therefore, we investigated the effect of a multispecies probiotic supplement on several markers of intestinal permeability, inflammation, and the quality of life in UC patients who were in clinical remission but still experienced various gastrointestinal complaints.

## 2. Materials and Methods

### 2.1. Study Design

The study was a twelve-week randomized double-blind placebo-controlled trial. All subjects were tested at baseline and after 6 and 12 weeks of intervention. At each time point, the subjects had to perform the 5-sugar absorption test, collect urine and faeces at home, visit the lab for a fasting blood sample, and fill out a questionnaire about disease-related quality of life (IBD-Q). In addition, patients filled out a diary that included the Bristol stool scale during the complete study period.

This study was conducted in accordance with the principles of the Declaration of Helsinki, approved by the medical ethical committee of Wageningen University, and registered at ClinicalTrials.gov (NCT02361957). Written informed consent was obtained from all study participants.

### 2.2. Study Population

Patients diagnosed with left-sided UC or pancolitis in clinical remission were recruited from the Hospital Gelderse Vallei (Ede, The Netherlands) database. Clinical remission was defined as having serum concentrations of C-reactive protein (CRP) of <10 mg/L, which was checked with a point-of-care CRP test, and calprotectin of <100 *µ*g/g, during their last medical check-up. Patients were excluded when having a history of GI surgery, diabetes mellitus, cancer, use of antibiotics during the last 3 months, current use of corticosteroids, alcohol consumption ≥21 servings a week for men and ≥14 for women, hypersensitivity to milk protein, gluten, or soy protein. Women who were currently pregnant or breast feeding were excluded as well. Mesalazine with a maximum dose of 2.4 g/day was the only medication for UC that was permitted during the study.

A total of 18 patients per group were required to detect a minimal difference in the lactulose : hamnose ratio of 0.03 with an SD of 0.01 [14], using a power of 80% and probability of 5%.

### 2.3. Intervention

Participants were equally and randomly assigned to the probiotics or placebo group. The randomization scheme was computer generated by Winclove using permuted blocks with a block size equal to 4. It was impossible for research personnel involved with participants to adjust randomization or discern what product participants were receiving, ensuring, true allocation concealment.

Patients used 2 sachets per day of 3 grams of the multispecies probiotic food supplement Ecologic® 825 or a placebo for twelve weeks, with a total concentration of 1.5 ∗ 10^10^ cfu/day. The same supplement had been shown to be effective in UC patients with pouchitis [24]. The supplement contained nine bacterial strains: *Bifidobacterium bifidum* W23, *Bifidobacterium lactis* W51, *Bifidobacterium lactis* W52, *Lactobacillus acidophilus* W22, *Lactobacillus casei* W56, *Lactobacillus paracasei* W20, *Lactobacillus plantarum* W62, *Lactobacillus salivarius* W24, and *Lactococcus lactis* W19, in a concentration of 2.5 ∗ 10^9^ colony-forming units (CFU) per gram. The placebo contained the same carrier ingredients (maize starch and maltodextrins) but no bacterial strains and was equal in appearance, smell, colour, taste, and package. As a marker of compliance, we counted the number of unused sachets people handed back in, and missed dosages reported in the diaries.

### 2.4. Measurements

During this study, a wide variety of methods was used to study the effects on markers of intestinal permeability, inflammation, and quality of life. Whole fasting venous blood samples were collected in L-heparin coated tubes and EDTA tubes, centrifuged for 10 minutes at 1550 rpm, and stored as serum and plasma samples at −80°C until analysis.

### 2.5. Intestinal Permeability

We used three tests for intestinal permeability: the 5-sugar absorption test in urine samples and the concentration of zonulin in serum and stool samples.

For the 5-sugar absorption test, subjects took a solution containing 1 g sucrose, 1 g lactulose, 1 g sucralose, 1 g erythritol, and 0.5 g L-rhamnose after an overnight fast at home. They refrained from foods and drinks until 2 hours after the sugar drink and collected 24 h urine in two separate urine containers: one for urine produced between 0 and 5 hours and one for urine produced between 5 and 24 hours. The urine containers were used to determine the 0–5 hours sucrose excretion, 0–5 hours lactulose/rhamnose (L/R) excretion ratio, and 5–24 hours sucralose/erythritol (5–24 h S/E) excretion ratio. The 24 hour sucralose/erythritol (24 h S/E) excretion ratio was determined by taking samples of both containers together.

After weighing the urine, 4 mL of both containers was sampled and stored at −20°C until analysis.

In the 5-sugar test, the collected urine samples are time bound as the sugar test solution passes through the different parts of the intestine over time. This method has been described extensively by van Wijck et al. [14, 16]. Low ratios of the urinary excretion of sugars indicate a low intestinal permeability, whereas high ratios indicate an increase in intestinal permeability [25]. To check for precision, we performed a recovery test as described in the literature [26]. Results were within the acceptable range.

Zonulin concentrations in faeces and serum were determined with an ELISA kit (Immundiagnostik AG, Bensheim, Germany) and measured with an ELISA plate reader at 450 nm against 620 nm as reference.

### 2.6. Inflammatory Markers

Several tests were performed to assess inflammation: C-reactive protein (CRP) and a number of cytokines were measured in blood samples, and calprotectin was measured in stool samples.

CRP was measured with the C-reactive protein (Latex) assay, a particle-enhanced turbidimetric description assay. In short, human CRP agglutinates with latex particles coated with monoclonal anti-CRP antibodies. The precipitate is determined turbidimetrically at 546 nm (Siemens Dimension Vista 1500).

For cytokine analysis, a multispot assay system was used (MSD® V-PLEX cytokine assay, Maryland, USA) and analysed on an MSD instrument (QuickPlex SQ 120 Instrument, Maryland, USA) [27]. Although a whole range of cytokines was measured (IFN*γ*, TNF*α*, IL-1b, IL-2, IL-4, IL-6, IL-10, IL-12p70, and IL-13), we only report on IFN*γ*, TNF*α*, IL-6, and IL-10. The other cytokines had 80% or more values below the detection limit.

Faecal calprotectin was measured with ELISA (CalproLab™, Calpro, Norway, represented by Selinion, the Netherlands) analysed at 405 nm.

Concentrations below the detection limit were not considered zero but were entered as half the value of the detection limit, which is a common procedure [28].

### 2.7. Quality of Life

To evaluate the symptoms, frequency, and stool type per day and possible changes in perceived wellbeing, subjects filled out a daily diary including the Bristol stool chart [29]. The irritable bowel disease questionnaire (IBD-Q) was filled out at baseline and after 6 and 12 weeks. Each question of the IBD-Q can have a score between 1 (“worst”) and 7 (“best”). The total score ranges from 32 to 224 with higher scores, indicating a better quality of life [30].

### 2.8. Statistical Analysis

We performed an intention-to-treat analysis. Statistical analysis was performed with SPSS (IBM SPSS Statistics, Version 22.0. Armonk, NY: IBM Corp.) and GraphPad Prism (Version 5.0 San Diego, CA). Normality of data was analysed with the Kolmogorov–Smirnov test. Differences between time points and between groups were tested by Student's *t*-tests or the Mann–Whitney U-test when the data were nonparametric. Correlations, based on baseline data, were calculated as Pearson's *r* for normally distributed variables and Spearman's rho for nonparametric distributions. A *p* value of <0.05 was considered statistically significant.

## 3. Results

### 3.1. Participant Characteristics

**Figure 1:**
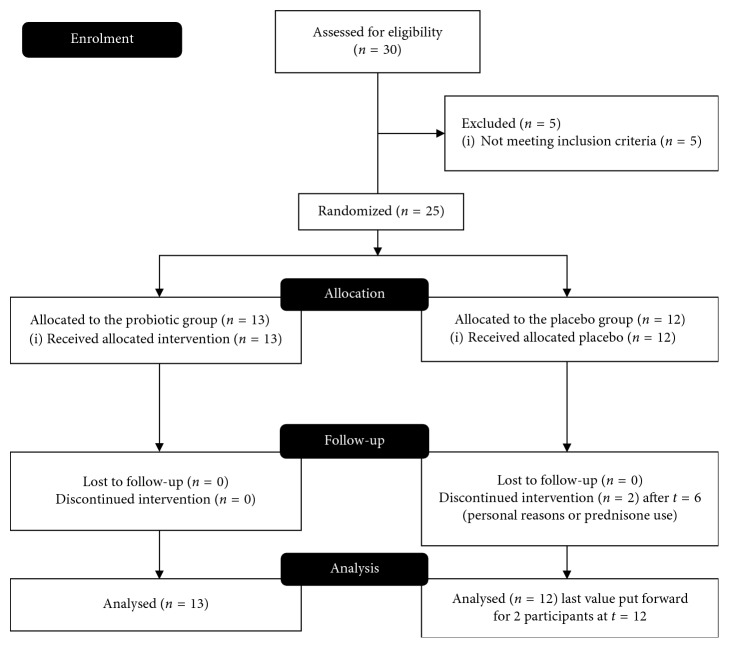
CONSORT flowchart for enrolment of participants and data analysis.

Twenty-five subjects, *n*=13 in the probiotic group and *n*=12 in the placebo group, were included, and 23 subjects completed all tests at 6 and 12 weeks. Gender distribution was 6 : 7 in the probiotic group and 7 : 5 in the placebo group. Mean age was (mean ± SD) 51.8 ± 13.3 in the probiotic group and 51.1 ± 11.9 in the placebo group. Subjects in the probiotics group took 77% of their supplements and subjects in the placebo group 84%. Two subjects had a low compliance (<50%) in the first 6-week period, but they were included in the intention-to-treat analysis. Two subjects in the placebo group dropped out; one because of prednisone use prescribed for a flare up and one because of personal reasons. Their week-6 values were put forward to week 12. A CONSORT flowchart for enrolment and analysis is presented in Figure 1.

### 3.2. Intestinal Permeability

**Figure 2:**
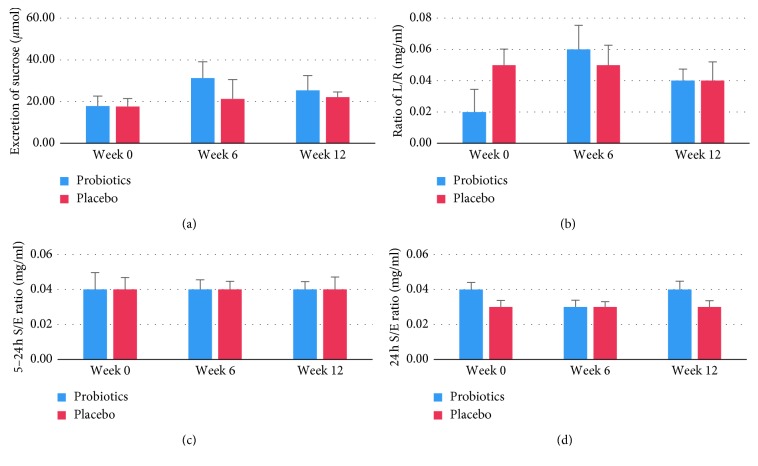
Results of the 5-sugar test with L/R, lactulose/rhamnose ratio, and S/E, sucralose/erythritol ratio. (a) Excretion of sucrose in *μ*mol; (b) L/R ratio; (c) 5–24 h S/E ratio; (d) 24 h S/E ratio. No significant differences between time periods or between groups were found. Values are means ± SD.

Data of the 5-sugar tests are shown in Figure 2 and Table 1. At baseline, permeability of the gastroduodenal unit was almost equal in the two groups, measured by a mean 17.7 *µ*mol/L urinary sucrose excretion in the first 5 hours of the test. Although sucrose excretion appeared to increase after 6 weeks and drop again after 12 weeks, none of the changes in time or differences between groups were significant. The L/R ratio varied between 0.02 and 0.06; none of the group or time effects were significant. The 5–24 h S/E ratio was 0.04 in both groups and remained stable throughout the whole intervention period. Slightly lower but again stable ratios were seen for the S/E ratios in the whole 24 h urine samples, with no significant differences between groups or in time. As could be expected, the 5–24 h S/E ratio and the 24 h S/E ratio were highly correlated (*r*=0.799, *p* < 0.01). Correlations between any of the other sugar absorption tests were weak (*r* < 0.16) and nonsignificant.

**Figure 3:**
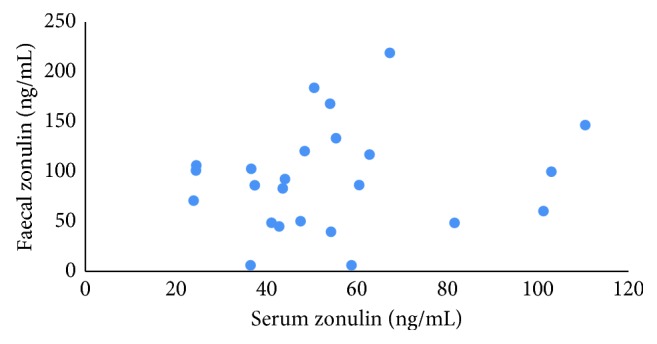
Correlation (*r*=0.16, *p*=0.456) between serum and faecal zonulin concentrations at baseline in CU patients in remission (*n*=24 paired samples).

**Figure 4:**
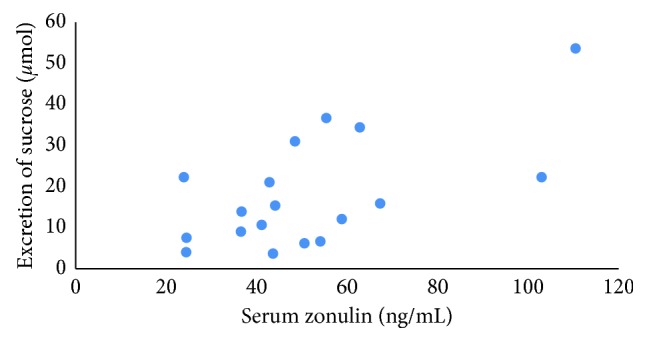
Correlation (*r*=0.62, *p*=0.006) between the 0–5 hour excretion of sucrose and serum zonulin concentrations at baseline in UC patients in remission (*n*=18 paired samples).

Serum zonulin at baseline was 58.3 ± 25.7 ng/mL in the probiotics group which was slightly higher than that in the placebo group (51.0 ± 22.2 ng/mL). In the probiotics group, serum zonulin dropped about 14% after 6 weeks and remained stable until the end of the study. In the placebo group, an initial decrease of 10% was followed by a return to baseline values after 12 weeks (Table 1). None of the group or time differences was significant. Faecal zonulin was close to 100 ng/mL throughout the study, with small and nonsignificant changes in time and between groups. In contrast to serum zonulin, which was highest at 6 weeks, faecal zonulin was lowest at 6 weeks. However, zonulin concentrations in serum were only weakly and nonsignificantly correlated with those in faeces at baseline (Figure 3) or at any other time point (data not shown) with a maximal Pearson's correlation of 0.19. We also tested whether the probiotics had an effect on total (serum + faecal) zonulin concentrations, but this was not the case. Serum zonulin, but not faecal zonulin, was significantly and moderately correlated with the excretion of sucrose in urine (*r*=0.62, *p* < 0.01, Figure 4) but not with the absorption and excretion of any of the other sugars. Similar results were found for the other time points (data not shown).

### 3.3. Inflammatory Markers

Overall, no effects of treatment or time on any of the inflammation markers were seen. At baseline, 2/13 patients in the probiotics group and 1/12 in the placebo group had CRP concentrations above the detection limit of 3 mg/L, and this hardly changed during the study period, so no treatment or time effects were seen (Table 1).

Most subjects had elevated baseline concentrations (≥3.80 pg/mL) of the proinflammatory cytokine IFN*γ*. Elevated concentrations were observed in 12/13 patients in the probiotic group and 10/12 patients in the placebo group. Even though concentrations of IFNγ increased in both groups during the study, these changes were not significant within or between the two groups. The concentrations of TNFα were elevated (≥0.74 pg/mL) in all but one patient: in 12/13 patients in the probiotics group and 12/12 in the placebo group [27]. However, the mean concentrations remained unchanged during the study. Also, for the cytokines IL-6 and IL-10, most patients showed higher values than the cutoffs: ≥0.29 pg/mL for IL-6 (12/13 in the probiotics group and 11/12 in the placebo group) and ≥0.21 pg/for IL-10 (9/13 in the probiotics groups and 11/12 in the placebo group). Again, changes in time were small and nonsignificant and not different for the two groups.

**Figure 5:**
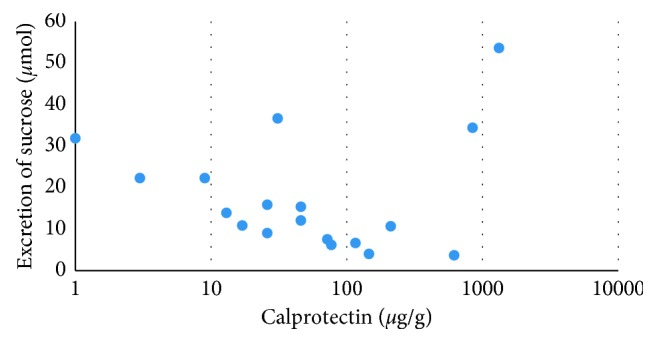
Correlation (*r*=0.55, *p*=0.014) between the 0–5 hour excretion of sucrose and calprotectin concentrations at baseline in UC patients in remission (*n*=18 paired samples).

**Figure 6:**
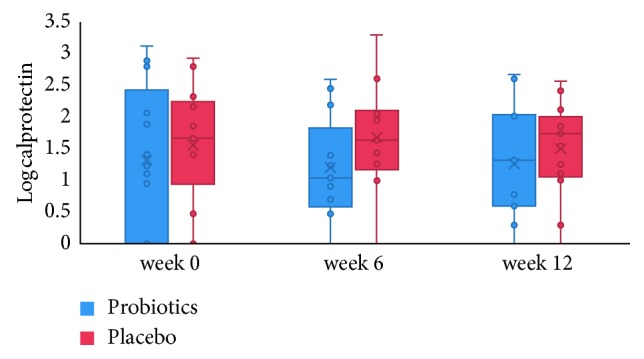
Faecal calprotectin (measured in *µ*g/g) during the 12-week intervention period in the probiotics and placebo group. The dotted line at log 2 reflects the cutoff for inflammation at 100 *µ*g/g [32].

Faecal calprotectin concentrations higher than the clinical cutoff value of 100 *µ*g/g [31, 32] were seen in about one-third of the patients in both groups and at all three time points (Figure 5). Although median concentrations of faecal calprotectin were higher in the placebo group, no significant time or group effects on median concentrations were found (Table 1). Calprotectin concentrations were significantly correlated with sucrose excretion (*r*=0.552, *p*=0.014) but not with any of the other measures (Figure 6).

Some of the inflammation markers were significantly correlated with one another: IL-6 and IFN*γ* (*r* = 0.64), IL-6 and IL-10 (*r* = 0.42), TNF*α* and IFN*γ* (*r* = 0.66), and TNF*α* and IL-6 (*r* = 0.59). However, no significant correlations were found between the cytokines and the more general inflammation markers CRP and calprotectin.

### 3.4. Quality of Life and Bowel Habits

Average baseline scores on the IBD-Q were 194 ± 22 for the probiotic group and 195 ± 21 for the placebo group. No treatment or time effect was seen on the scores for the IBD-Q. We also calculated the scores per category: bowel, systemic, emotion, and social, but again no significant differences were found.

**Figure 7:**
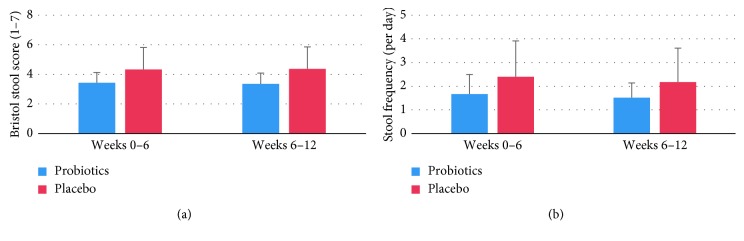
Average stool type according to the 7-points Bristol stool scale (a) and number of stools per day (b) in patients using probiotics (*n*=13) or placebo (*n*=12).

Consumption of the probiotic supplement did not affect the number of stools per day or the faecal consistency as measured by the Bristol stool chart. The score for the Bristol stool type in the probiotic group was 3.4 over the whole 12-week period, against 4.3 in the first 6 weeks and 4.2 in weeks 6–12 in the placebo group (Figure 7). Stool frequency was slightly less than 2 per day in the probiotics group and slightly more than 2 in the placebo group, but differences between groups or changes in time were not significant. We also tested whether more subjects in the probiotics group attained a “normal” stool type and frequency over the 12-week period, as defined by a Bristol stool type of 3, 4, or 5 at a frequency of more than 3 times per week, but less than 4 times per day [33]. Although this number was higher in the probiotics group (9/13) than in the placebo group (6/12), this difference was not significant.

## 4. Discussion

In this study, intestinal permeability, inflammation, bowel habits, and quality of life were not significantly affected by daily use of a multispecies probiotic in UC patients in remission. Overall, hardly any changes in the outcomes were seen over time, confirming that these patients were in a stable phase of their disease.

Interesting correlations were found between different measurements which were performed during the study. Urinary sucrose secretion was correlated with serum zonulin levels and with faecal calprotectin levels, whereas all other parameters were not correlated. Sucrose is used to measure gastroduodenal permeability since this disaccharide is rapidly hydrolysed in the duodenum [14]. Calprotectin is an abundant neutrophil protein that is released during inflammation [34]. It is often used in the diagnosis and monitoring of IBD patients. Zonulin is a modulator of tight junction proteins [35]. An earlier study has shown a correlation between serum zonulin levels and lactulose/mannitol ratio in patients with type 1 diabetes [36]. In contrast, a study with inulin-enriched pasta in healthy volunteers showed no significant correlation between serum zonulin levels and lactulose/mannitol ratio, although on a group level both were decreased compared to controls [37]. In our study, also no correlation was found between serum zonulin and lactulose/rhamnose ratio, similar of our previous findings in a group of migraine patients [38] As we did now find a correlation between serum zonulin and sucrose, zonulin might be a marker for gastroduodenal permeability rather than whole small intestinal permeability.

No correlation was found between serum and faecal zonulin levels, which is in agreement with other studies [38, 39]. Both serum and faecal zonulin levels have been used previously to measure intestinal permeability. As we found no correlation of faecal zonulin levels with any of the other markers measured, further research is needed to validate the relevance of this marker. The use of serum levels of zonulin as a marker of intestinal permeability also needs to be used in some patient populations with caution, as for instance the serum levels seem to be correlated with liver function [40, 41] in case of liver diseases.

Overall it is important to realize that many factors contribute to mucosal barrier function, including epithelial integrity [6]. Different markers for intestinal permeability can reflect different processes, like paracellular flux, epithelial integrity, and/or or bacterial translocation, so interpretation should be done in context of the disease and what is exactly measured [42].

No effect of the probiotic intervention of the measured markers of gut permeability was found. Even a more sophisticated statistical analysis using a principal response curve showed that there was no clear intervention effect on intestinal permeability or inflammatory markers (data not shown). We do not think that we missed an effect due to the type or dose of the probiotics or the study length: we chose a probiotic mixture that had been used in other study populations with positive results: it restored mucosal barrier function in patients with pouchitis [24] and relieved gastrointestinal complaints in otherwise healthy volunteers [8]. Our dose was comparable to that in studies that showed significant results with probiotics [24, 43–45]. Moreover, the intervention period of 3 months should be long enough to establish effects on intestinal barrier function; positive studies with probiotics had a mean duration on 40 days [8].

A more likely explanation for not finding an effect might be the choice of the patient population; all were in remission and in a mild state of the disease. This was, for example, reflected in their faecal calprotectin concentrations, which were low in both groups. Although these concentrations were different at baseline, they were not clinically relevant. We think it is plausible that the stable disease conditions made intestinal permeability and inflammation markers unresponsive to treatment, but we could not find evidence in the literature for this hypothesis. However, in a study with healthy volunteers and a challenge with indomethacin, no effects of the probiotics could be found [46]. The stable phase of the disease was reflected in the results of the novel 5-sugar absorption test, which provides insights into permeability in segments of the gut, in contrast to the traditionally used lactulose/mannitol sugar test [16]. The sugar ratios we found were comparable to those of other subjects without inflammation: patients with irritable bowel syndrome and healthy controls [47] and healthy volunteers that were treated with indomethacin [14]. It might be that the test is more suitable for finding moderate to large differences between patient groups than for finding subtle intervention effects. Because some of the sugars used in the test can be found in the diet, standardizing the meal on the evening before and fasting for the first 5 hours after starting the test may improve the accuracy of the test. Also, it seems prudent to check the completeness of the urine collection with a recovery marker, especially for the concentration of sucrose in the first 5 h batch.

Finding patients that fulfill the inclusion criteria and were willing to take part proved to be hard, and as a result, only 70% of the original sample size was recruited. However, the results do not suggest that more subjects would have given significant results. The sample size was based on a difference in the L/R ratio of 0.03 with an SD of 0.01 [14], whereas in our study, we found a much smaller difference and a much larger SD, which means that even the original sample size would not have been enough to detect the hypothesized changes as statistically significant.

The remission state was also shown by the cytokines levels found in the study subjects, which were similar to those reported in the literature for UC patients in remission. Although there is no clear serum cytokine profile that is typical for UC, several studies have reported increased concentrations of the proinflammatory cytokines IL-6, TNF*α*, and IFN*γ*, while the results on the anti-inflammatory cytokine IL-10 are less consistent [48]. In our study, IFN*γ*, TNF*α*, and IL-6 were indeed increased in the majority of subjects, which is in line with the literature. The anti-inflammatory cytokine IL-10 was increased in the majority of subjects in our study, which is in line with a recent study showing that UC patients who had IBS-like symptoms while being in remission, similar to our subjects, had higher IL-10 concentrations in serum than patients who did not have these symptoms [49].

The study was not powered or long enough to test for differences in flare-ups during the remission period. However, the one flare-up during the study was in the placebo group, suggesting that a larger and longer study with the same probiotic may be prudent.

Despite the lack of effect, this study has value for future studies because it is the first to combine multiple tests related to intestinal permeability, inflammation markers, and perceived wellbeing. We had expected to see closer correlations between these outcomes, or between changes in outcomes, but we found only few. Of the three markers for intestinal permeability, we only found good correlation between serum zonulin and sucrose excretion in the first 5 hours after intake. Faecal zonulin showed a much lower correlation. This may indicate that serum zonulin is a better indicator of intestinal permeability than faecal zonulin, but this finding remains to be confirmed in other studies.

## 5. Conclusion

In this study, with UC patients who did not have an increased intestinal permeability, no short-term benefits of a multispecies probiotic were seen. Further studies should include patients with increased permeability. Longer study duration is necessary to investigate the possible effects of probiotics on prevention of a relapse. Because of its correlation with sucrose excretion in the small intestine, we suggest to use serum zonulin rather than faecal zonulin as a measure of intestinal permeability in future studies.

## Figures and Tables

**Table 1:** Intestinal permeability and inflammation measured in urine, faeces, and serum samples at three time points during the study.

	Probiotic group (*n*=13)			Placebo group (*n*=12)		
	Week 0	Week 6	Week 12	Week 0	Week 6	Week 12
Urinary sucrose excretion (*μ*mol)	17.8 ± 15.2	31.2 ± 23.8	25.4 ± 18.3	17.6 ± 11.4	21.3 ± 28.3	22.1 ± 28.3
Urinary L/R ratio	0.02 ± 0.02	0.06 ± 0.05	0.04 ± 0.04	0.05 ± 0.03	0.05 ± 0.04	0.04 ± 0.03
Urinary S/E 5–24 h ratio	0.04 ± 0.03	0.04 ± 0.02	0.04 ± 0.01	0.04 ± 0.02	0.04 ± 0.02	0.04 ± 0.02
Urinary S/E 24 h ratio	0.04 ± 0.01	0.03 ± 0.01	0.04 ± 0.01	0.03 ± 0.01	0.03 ± 0.01	0.03 ± 0.01
Serum zonulin (ng/mL)	58.3 ± 25.7	50.0 ± 17.4	49.6 ± 23.6	51.0 ± 22.2	45.8 ± 9.7	51.8 ± 17.9
Faecal zonulin (ng/mL)	95.6 ± 52.3	113.1 ± 69.1	89.6 ± 64.7	91.0 ± 53.3	127.1 ± 100.9	118.4 ± 91.9
C-reactive protein (*n* > 3 mg/mL)	2/13	0/13	2/13	1/12	2/12	1/12
IFN*γ* (pg/mL)	11.5 ± 7.0	17.4 ± 15.3	42.2 ± 42.5	8.4 ± 4.9	12.7 ± 8.8	19.3 ± 9.4
IL-10 (pg/mL)	0.5 ± 0.7	0.4 ± 0.7	0.4 ± 0.5	0.6 ± 0.9	0.8 ± 1.4	0.3 ± 0.1
IL-6 (pg/mL)	0.8 ± 0.8	0.7 ± 0.6	0.8 ± 0.5	0.7 ± 0.4	0.7 ± 0.5	0.8 ± 0.4
TNF*α* (pg/mL)	2.2 ± 0.8	2.2 ± 0.8	2.3 ± 0.7	2.2 ± 0.6	2.3 ± 0.6	2.0 ± 0.4
Calprotectin (*µ*g/g)	17.0	11.0	21.0	46.0	65.5	56.0

L/R: lactulose/rhamnose ratio; S/E: sucralose/erythritol ratio. No significant differences between time periods or between groups were found. All values are in mean ± SD except for CRP (fraction) and calprotectin (median).

## Data Availability

The raw data used to support the findings of this study may be released upon application to Wageningen University, Division of Human Nutrition, which can be contacted at carrie.wegh@wur.nl.
